# Development of a Simple and Robust Whole Blood Assay with Dual Co-Stimulation to Quantify the Release of T-Cellular Signature Cytokines in Response to *Aspergillus fumigatus* Antigens

**DOI:** 10.3390/jof7060462

**Published:** 2021-06-08

**Authors:** Chris D. Lauruschkat, Lukas Page, P. Lewis White, Sonja Etter, Helen E. Davies, Jamie Duckers, Frank Ebel, Elisabeth Schnack, Matthijs Backx, Mariola Dragan, Nicolas Schlegel, Olaf Kniemeyer, Axel A. Brakhage, Hermann Einsele, Juergen Loeffler, Sebastian Wurster

**Affiliations:** 1 Department of Internal Medicine II, University Hospital of Wuerzburg, 97080 Wuerzburg, Germany; Lauruschka_c@ukw.de (C.D.L.); E_Page_L@ukw.de (L.P.); sonja.etter@stud-mail.uni-wuerzburg.de (S.E.); Einsele_H@ukw.de (H.E.); 2 Public Health Wales, Microbiology Cardiff, Wales CF14 4XW, UK; lewis.white@wales.nhs.uk (P.L.W.); Matthijs.backx2@wales.nhs.uk (M.B.); 3 Department of Respiratory Medicine, University Hospital of Wales, Llandough Site, Cardiff CF64 2XX, UK; Helen.Davies30@wales.nhs.uk; 4 Cystic Fibrosis Unit, University Hospital of Wales, Llandough Site, Cardiff CF64 2XX, UK; Jamie.Duckers@wales.nhs.uk; 5 Institute for Infectious Diseases and Zoonoses, Ludwig-Maximilians-University of Munich, 80539 Munich, Germany; Frank.Ebel@micro.vetmed.uni-muenchen.de (F.E.); Elisabeth.Schnack@micro.vetmed.uni-muenchen.de (E.S.); 6 Department of Surgery I, University Hospital of Wuerzburg, 97080 Wuerzburg, Germany; Dragan_M@ukw.de (M.D.); schlegel_n@ukw.de (N.S.); 7 Department of Molecular and Applied Microbiology, Leibniz Institute for Natural Product Research and Infection Biology–Hans-Knöll-Institute (HKI), 07745 Jena, Germany; olaf.kniemeyer@hki-jena.de (O.K.); axel.brakhage@leibniz-hki.de (A.A.B.); 8 Department of Infectious Diseases, Infection Control and Employee Health, The University of Texas MD Anderson Cancer Center, Houston, TX 77030, USA

**Keywords:** immunoassay, biomarker, *Aspergillus*, cytokines, inflammation, adaptive immunity

## Abstract

Deeper understanding of mold-induced cytokine signatures could promote advances in the diagnosis and treatment of invasive mycoses and mold-associated hypersensitivity syndromes. Currently, most T-cellular immunoassays in medical mycology require the isolation of mononuclear cells and have limited robustness and practicability, hampering their broader applicability in clinical practice. Therefore, we developed a simple, cost-efficient whole blood (WB) assay with dual α-CD28 and α-CD49d co-stimulation to quantify cytokine secretion in response to *Aspergillus fumigatus* antigens. Dual co-stimulation strongly enhanced *A. fumigatus*-induced release of T-cellular signature cytokines detectable by enzyme-linked immunosorbent assay (ELISA) or a multiplex cytokine assay. Furthermore, T-cell-dependent activation and cytokine response of innate immune cells was captured by the assay. The protocol consistently showed little technical variation and high robustness to pre-analytic delays of up to 8 h. Stimulation with an *A. fumigatus* lysate elicited at least 7-fold greater median concentrations of key T-helper cell signature cytokines, including IL-17 and the type 2 T-helper cell cytokines IL-4 and IL-5 in WB samples from patients with *Aspergillus*-associated lung pathologies versus patients with non-mold-related lung diseases, suggesting high discriminatory power of the assay. These results position WB-ELISA with dual co-stimulation as a simple, accurate, and robust immunoassay for translational applications, encouraging further evaluation as a platform to monitor host immunity to opportunistic pathogens.

## 1. Introduction

Humans inhale hundreds of airborne conidia of the ubiquitous mold *Aspergillus fumigatus* daily, resulting in constant activation of the innate and adaptive immune system in order to clear the fungus from the lungs and maintain protective tolerance [1,2,3]. Imbalance of immunity can lead to a wide spectrum of *A. fumigatus*-associated diseases, ranging from invasive aspergillosis to hypersensitivity disorders [2]. As fine-tuning of the anti-*Aspergillus* response by T-cellular cytokines has major implications for protective immunity, recovery from invasive infection, and inflammatory immunopathology, deeper understanding of cytokine signatures may lead to advances in the diagnosis and treatment of invasive mycoses and mold-associated hypersensitivity syndromes [1,4,5,6].

Currently available investigational assays to quantify and analyze mold-specific T cells mostly rely on peripheral blood mononuclear cell (PBMC)-based flow cytometry or measurement of cytokine release via enzyme-linked immunospot (ELISPOT) and enzyme-linked immunosorbent assays (ELISA) [7,8,9,10,11]. While showing potential as novel supportive biomarkers in medical mycology [7,8,9,10,11], PBMC-based assays have crucial drawbacks limiting their precision, robustness, and feasibility in a clinical setting [8,12,13]. These limitations include varying reproducibility, difficult protocol standardization, significant costs and hands-on-time, and the considerable impact of pre-analytical sample handling [12,13,14].

To overcome these drawbacks, there is a growing interest in the development of whole blood (WB)-based protocols to determine T-cell responses to fungal antigens [15,16], following the clinical success of WB-based tests in tuberculosis and cytomegalovirus (CMV) diagnostics and research [17,18,19]. Although WB assays are more robust than PBMC-based protocols [16], concerns about impaired test performance remain when stimulation conditions are suboptimal, e.g., in the presence of immunosuppressive therapy or after long pre-analytic delays [16,20,21,22,23]. Furthermore, most functional T-cell assays currently applied in the clinical routine are designed to specifically detect interferon gamma (IFN-γ) [24,25,26,27] and thus require optimization for reliable analysis of the complex cytokine milieu elicited by mold antigens. Prior work suggested differential robustness of individual T-helper cell subsets, with particularly poor reliability of type 17 T-helper cell (Th17) stimulation [28] and, consequently, quantification of IL-17 [12], a cytokine regarded as a cornerstone in both protective anti-*Aspergillus* immunity and inflammatory immunopathology [1].

Although not commonly employed in commercially available WB-ELISA kits and IFN-γ release assays (IGRAs), previous studies have highlighted that co-stimulatory antibodies can considerably increase the detection efficacy and robustness of functional T-cell readouts [29,30]. Specifically, we have shown in two recent studies that enhanced co-stimulation improves (flow cytometric) detection rates of mold-reactive T-cells while attenuating the detrimental impact of immunosuppressive pretreatment on assay performance [16,23]. Building upon these advances, we herein developed and tested a simple, cost-efficient WB-ELISA system with enhanced co-stimulation to quantify T-cellular signature cytokines in response to *A. fumigatus* antigens.

## 2. Materials and Methods

### 2.1. Blood Collection

Venous blood was obtained using Monovette® lithium heparin blood collection tubes (Sarstedt, Nümbrecht, Germany). Exclusion criteria for healthy blood donors (age 18–50, 16 male, 7 female) were acute infections, pregnancy, as well as recent immunomodulatory or antimicrobial therapy. For studies in patients with chronic lung diseases, 2 patients with cystic fibrosis (CF) and elevated *Aspergillus*-specific immunoglobulin E (age 41–47, 1 male, 1 female), 3 patients with allergic bronchopulmonary aspergillosis (ABPA, age 33–83, 1 male, 2 female), 4 patients with chronic pulmonary aspergillosis (CPA, age 60–80, 3 male, 1 female), and 5 control patients with non-mold-related interstitial lung diseases (age 56–90, 4 male, 1 female) were enrolled at the University Hospital of Wales.

### 2.2. Generation of Aspergillus fumigatus mycelial lysate (AfuLy)

**Figure 1:**
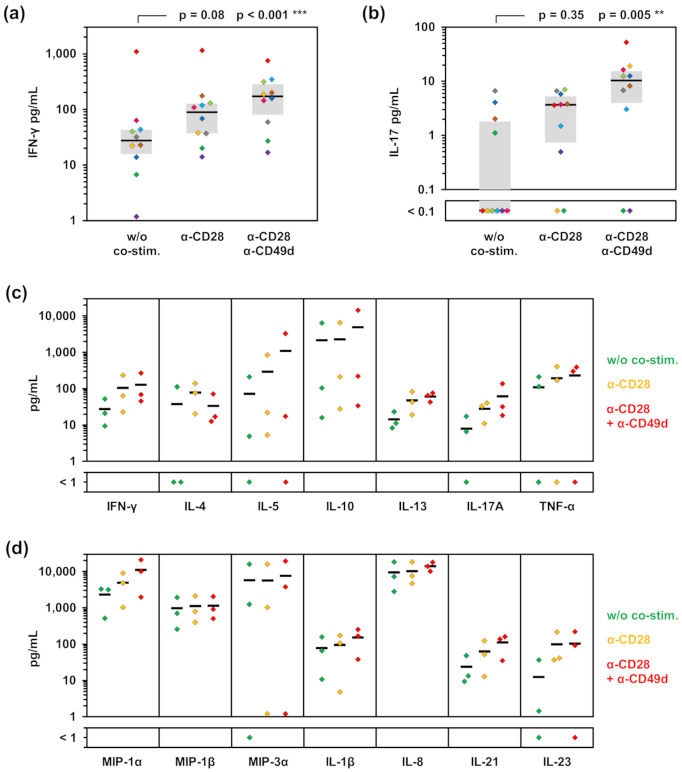
Impact of co-stimulatory factors on *A. fumigatus* lysate-induced cytokine secretion. (**a**,**b**) WB from 10 highly mold-exposed subjects, as defined in References [3,33], was stimulated for 24 h with AfuLy in RPMI-supplemented test tubes containing α-CD28, α-CD28 plus α-CD49d, or no co-stimulatory factors (w/o co-stim.). IFN-γ (**a**) and IL-17 (**b**) concentrations were quantified in plasma supernatants by ELISA. For all results shown, the unspecific background, determined in a test tube containing the respective co-stimulation cocktail but no lysate, was deduced from antigen-induced cytokine concentrations. Corresponding test results from each donor are indicated by the same color. The Friedman test and Dunn’s multiple comparison test were used for significance testing. (**c**,**d**) WB samples from 3 additional healthy donors were stimulated as described above. Cytokine concentrations in plasma supernatants were quantified using a 21-plex Luminex assay. Individual background-adjusted concentrations of T-cellular signature cytokines (**c**) and selected cytokines predominantly produced by antigen-presenting cells (**d**) are shown. Black bars indicate means.

To generate *A. fumigatus* (ATCC46645) protein extracts, conidia (1 × 10^6^/mL) were inoculated in *Aspergillus* minimal medium [31] and cultured at 37 °C, 200 rpm, in shake flasks. Mycelium was harvested after 20 h by filtration through Miracloth (Merck Millipore Calbiochem, Darmstadt, Germany). Subsequently, frozen mycelium was ground in liquid nitrogen by using a mortar and pestle and resuspended in sterile PBS (approximately 100 mg ground mycelium in 1 mL of PBS). The crude extract was centrifuged for 15 min at 20,000 g. Thereafter, the supernatant was transferred to a new microcentrifuge tube and stored at −80 °C until use. The protein concentration was determined using the infrared-based Direct Detect System (Merck Millipore, Darmstadt, Germany). For selected experiments (Figures 1 and 5), a formerly commercially available lysate (Miltenyi Biotec, Bergisch Gladbach, Germany) was used instead.

### 2.3. Generation of Recombinant Aspf4

For recombinant expression of the *A. fumigatus* allergen Aspf4, the respective sequence (Afu2g03830, obtained from the *Aspergillus* Genome Database, http://aspgd.org/, accessed on 13 June 2019) was amplified from cDNA derived from *A. fumigatus* strain AfS35. PCR amplification was performed using Q5 High-fidelity DNA polymerase (New England Biolabs, Frankfurt am Main, Germany). The following oligonucleotide sequences were used: GCAGGATCCCACGAGCGCCGCCACCTCCAC (forward, BamHI restriction site underlined) and GCAAAGCTTCTACTCCTTGTAGTCGAGGTT (reverse, HindIII restriction site underlined). The PCR amplicons were cloned into the expression vector pQE30 (Qiagen, Hilden, Germany) using the primer-derived restriction sites. After transformation into *E. coli* strain M15 pREP4 (Qiagen, Hilden, Germany), the recombinant proteins were purified using HisTalon gravity columns (Takara-Bio Inc., Kusatsu, Präfektur Shiga, Japan) according to the vendor’s instructions.

### 2.4. Quality Control of Lysates and Aspf4 Antigen

Endotoxin concentrations in lysates and Aspf4 antigen preparations were determined using the Endochrome-K assay kit and EndoScan V software 4.0 (SP1) (Charles River Wilmington, MA, USA) according to the manufacturer’s manual. A very conservative cut-off for endotoxin concentrations (1 endotoxin unit per mg of antigen) was applied to preclude an influence of endotoxin contamination on test results.

### 2.5. Preparation of WB Stimulation Tubes

Anticoagulant-free blood collection tubes (Sarstedt, Nümbrecht, Germany) were prepared with Roswell Park Memorial Institute medium (RPMI, Gibco, Thermo Fisher Scientific, Waltham, MA, USA) or CTL Test^TM^ medium (CTL Europe, Bonn, Germany) supplemented with co-stimulatory antibodies (Miltenyi Biotec, Bergisch Gladbach, Germany) and antigenic stimuli, as outlined in Table 1. The unstimulated background control contained co-stimulatory factors and medium, but no stimulus. The positive control consisted of medium and phytohemagglutinin (PHA, Sigma-Aldrich, St. Louis, MO, USA). Stimulation tubes were cryopreserved at –20 °C for up to four weeks. 

### 2.6. WB Stimulation and Quantification of Cytokine Concentrations in Plasma Supernatants

Stimulation tubes were thawed and brought to RT before use. Within 90 min of the blood draw (up to 6 h for CF/ABPA/CPA patients), 500 µL WB was injected into each stimulation tube with a graduated 1 mL insulin syringe. Stimulation tubes were inverted 10 times and incubated for 24–26 h at 37 °C. Plasma was collected after a 20 min centrifugation step at 2000 g and cryopreserved at −20 °C. Cytokine concentrations were determined using IFN-γ and IL-17 ELISA Max Deluxe Sets (Biolegend, San Diego, CA, USA) following the manufacturer’s instructions. Absorbance was read on a NanoQuant Infinite 200M Pro microplate reader (Tecan, Maennedorf, Switzerland) and cytokine concentrations were interpolated from a 7-point standard curve after subtraction of baseline absorbance in a plain medium control. The Milliplex® MAP human high-sensitivity T Cell magnetic bead panel kit (Merck, Darmstadt, Germany) was used for the multiplex cytokine assay. Cytokine concentrations were determined using a Luminex200 reader (Luminex Austin, TX, USA) in combination with the XPONENT3 (Luminex Austin, TX, USA) and Milliplex Analyte software (Merck, Darmstadt, Germany).

### 2.7. T-Cell Depletion of WB Samples

The Whole Blood Column Kit (Miltenyi Biotec, Bergisch Gladbach, Germany) was applied to a MidiMACS™ cell separator (Miltenyi Biotec, Bergisch Gladbach, Germany) and rinsed with PBS (Sigma-Aldrich, St. Louis, MO, USA) supplemented with 0.5% human AB serum (Sigma-Aldrich, St. Louis, MO, USA). WB samples were incubated with StraightFrom^®^ Whole Blood CD3 MicroBeads (Miltenyi Biotec, Bergisch Gladbach, Germany; 50 µL bead suspension per mL of WB) for 10 min at room temperature and added to the columns. For mock depletion, no CD3 MicroBeads were added. Thereafter, CD3 depleted and non-depleted blood was injected into the stimulation tubes as described above.

### 2.8. Flow Cytometry

WB samples were stimulated at 37 °C as described above for a total of 6 h for granulocyte and NK-cell stimulation and 24 h for T-cell stimulation. For NK-cell and T-cell analysis, brefeldin A (10 μg/mL, Sigma-Aldrich St. Louis, MO, USA), BD GolgiStop™ (0.67 µL/mL, BD Biosciences, San Jose, CA, USA), and CD107a-PE-Vio770 (10 µL/mL, Miltenyi, Bergisch Gladbach, Germany) were added to the stimulation tubes after 2 and 4 h, respectively. After incubation, cells were processed and stained as described before [16]. Fluorescent antibodies used in this study are listed in Supplementary Table S1. Cells were analyzed using a Cytoflex AS34240 flow cytometer (Beckman Coulter, Brea, CA, USA). Downstream data analysis was performed with Kaluza v2.1 (Beckman Coulter, Brea, CA, USA).

### 2.9. Statistics

Antigen-specific cytokine release was determined by subtracting the cytokine concentrations in the unstimulated control tube from antigen-stimulated cytokine concentrations. All cytokine concentrations were then normalized per mL of the subject’s blood volume injected into the stimulation tubes. To determine coefficients of variation (CVs), standard deviations were divided by mean values. In line with published recommendations [32], target CVs were 25% for well-defined antigens (Aspf4) and 35% for less well-defined antigens (AfuLy). GraphPad Prism v8 and Microsoft Excel were used for data analysis. Applicable significance tests are specified in the figure legends. Significance levels are denoted by asterisks: * *p* < 0.05, ** *p* < 0.01, and *** *p* < 0.001.

## 3. Results

### 3.1. Impact of Co-Stimulatory Factors on Aspergillus-Induced Cytokine Release

At first, we compared the stimulation efficacy of AfuLy without co-stimulation, with α-CD28, or α-CD28 combined with α-CD49d. Although not reaching statistical significance, α-CD28 alone resulted in 3.2-fold higher median IFN-γ response to the lysate compared with non-co-stimulated samples and was essential to elicit detectable IL-17 release in the majority of subjects tested (Figure 1a,b). Dual co-stimulation further increased median *A. fumigatus*-induced IFN-γ and IL-17 concentrations and led to enhanced release in 17 out of 20 individual measurements compared with both the non-co-stimulated protocol and α-CD28 alone (Figure 1a,b). In contrast, no influence of co-stimulation on background IFN-γ and IL-17 secretion (median 0–1 pg/mL for both cytokines and all 3 conditions) was seen in control samples not containing AfuLy.

To corroborate the impact of dual co-stimulation on a broader selection of cytokines and to confirm our findings with a different readout methodology, we used a 21-plex Luminex assay (Figure 1c,d). Mean concentrations of all studied T-helper cell signature cytokines except IL-4 were increased in samples with dual co-stimulation versus α-CD28 alone, with the strongest enhancement found for IL-5 and IL-17A (Figure 1c). Interestingly, concentrations of selected cytokines derived from antigen-presenting cells (APCs), especially MIP-1α and IL-21, were also increased in plasma supernatants from tubes with dual co-stimulation (Figure 1d).

### 3.2. T-Cell Dependency of Immune Cell Stimulation

**Figure 2:**
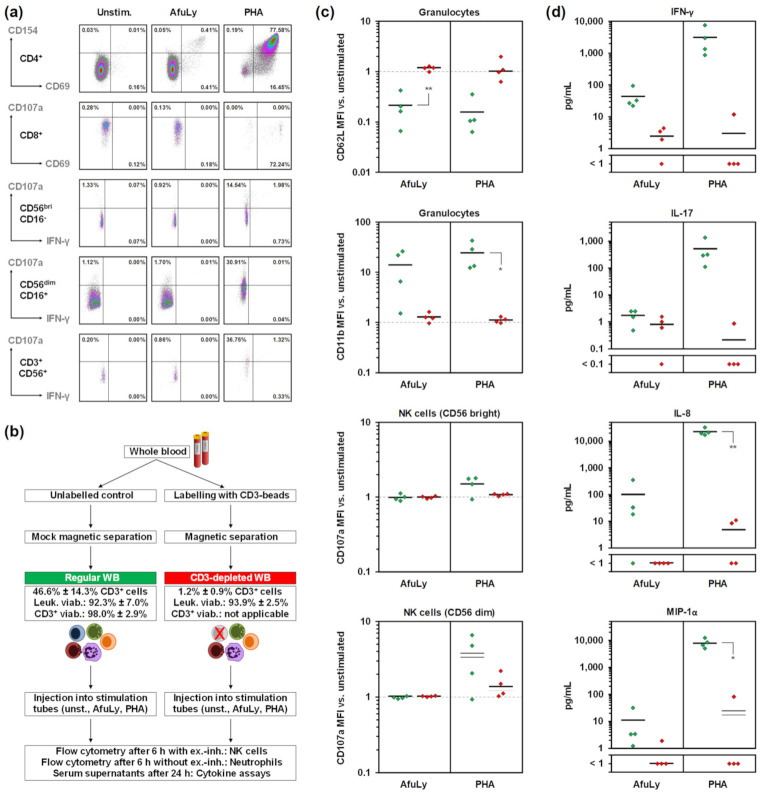
T-cell-dependent immune cell activation and cytokine release in WB-ELISA stimulation tubes. (**a**) Representative flow cytometry panels showing the expression of key activation markers (CD69, CD154, and/or CD107a) and IFN-γ by unstimulated, AfuLy-stimulated, and PHA-stimulated CD4^+^ T-helper cells, CD8^+^ cytotoxic T-cells, cytokine-producing CD56^bright^CD16^−^ NK cells, cytotoxic CD56^dim^CD16^+^ NK cells, and CD3^+^CD56^+^ NKT cells, as determined by intracellular staining. (**b**) Flowchart of experimental procedures to determine the T-cell dependency of activation marker expression and cytokine response by comparison of regular and CD3-depleted WB. Ranges of CD3^+^ cell frequencies among leukocytes and leukocyte viability after magnetic bead-based CD3 depletion and mock depletion are provided. (**c**) Relative expression of the degranulation marker CD62L and activation marker CD11b on granulocytes and CD107a expression of NK cells upon AfuLy and PHA stimulation of regular (green) and CD3-depleted (red) WB, normalized to unstimulated cells. (**d**) Background-adjusted concentrations of IFN-γ, IL-17, IL-8, and MIP-1α (CCL3) in plasma supernatants of regular (green) and CD3-depleted (red), AfuLy- and PHA-stimulated WB. (**c**,**d**) Paired two-sided t-test; *n* = 4. Black bars indicate means. Abbreviations: AfuLy = *A. fumigatus* mycelial lysate, ex.-inh. = exocytosis inhibitors, Leuk. = leukocyte, MFI = mean fluorescence intensity, PHA = Phytohaemagglutinin, unst. = unstimulated, viab. = viability, WB = whole blood.

Next, we performed a more detailed assessment of AfuLy-induced leukocyte activation in our WB-ELISA stimulation system. As extensively documented elsewhere [16,23], AfuLy-stimulation of WB with dual co-stimulation facilitates sensitive detection of *Aspergillus*-reactive T-helper cells by intracellular staining of activation markers (e.g., CD69 or CD154), even in healthy donors with moderate mold exposure (representative donor shown in Figure 2a). Importantly, activation of natural killer (NK) cells and natural killer T (NKT) cells by AfuLy was minimal, as evidenced by minor changes in CD107a and IFN-γ expression compared with unstimulated cells (Figure 2a–c). These results suggest that NK cells and NKT cells are not major confounders of IFN-γ quantification in our system. 

In order to further verify T-cell specificity of antigen-induced leukocyte activation and cytokine release, we compared the response of regular and T-cell-depleted WB to AfuLy and PHA (Figure 2b). T-cell depletion by magnetic separation of CD3^+^ T cells was highly effective (>95% reduction in CD3^+^-cell content) and leukocyte viability remained high (Figure 2b). Of note, both AfuLy and PHA elicited activation (CD11b upregulation) and degranulation (loss of CD62L) of granulocytes; however, these responses were strongly dependent on the presence of T-cells (Figure 2c). In contrast to AfuLy, PHA was able to induce CD107a upregulation in both CD16^bright^ and CD16^dim^ NK cells, albeit NK-cell activation was largely T-cell-dependent (Figure 2c). 

Expectedly, depletion of CD3 cells markedly lowered the release of T-helper-cell signature cytokines IFN-γ and IL-17 in response to AfuLy and PHA (Figure 2d). Although not reaching significance due to inter-individual baseline variation in cytokine release, these data further underscore the T-cell specificity of these cytokines in our assay system. Notably, CD3-depletion also strongly abrogated the AfuLy- and PHA-induced release of IL-8 and MIP-1α (CCL3), key indicator cytokines for the activation of neutrophils and mononuclear phagocytes [34,35,36,37]. Altogether, these results suggest that WB-ELISA with dual co-stimulation captures both *A. fumigatus*-induced T-cell activation and T-cell/APC feedback loops, while providing a highly T-cell-dependent stimulation environment.

### 3.3. Evaluation of Assay Reproducibility

**Figure 3:**
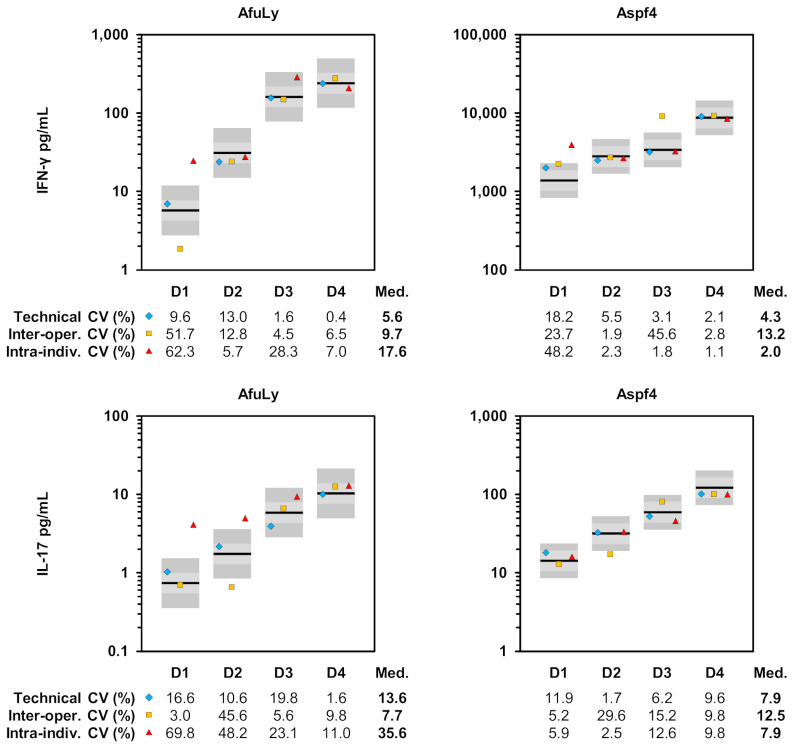
Technical and intra-individual variation of WB-ELISA. WB samples from 4 highly mold-exposed healthy subjects were stimulated with AfuLy or Aspf4 using RPMI-supplemented, α-CD28-, and α-CD49d-containing stimulation tubes as described in the Materials and Methods Section. After 24 h of stimulation, IFN-γ and IL-17 concentrations in plasma supernatants were quantified by ELISA. Individual results are indicated by black bars. For each donor and antigen (including the unspecific background control), a second set of stimulation tubes was prepared by the same operator using the same blood sample to determine technical variation (light blue diamonds). In addition, another set of stimulation tubes was injected by a different operator using blood from the same venipuncture to determine the inter-operator variation (yellow squares). Another blood draw was performed from the same subjects after a period of at least 4 weeks and cytokine concentrations (red triangle) were compared with the initial measurement (black bar) to calculate the intra-individual variation. All donors had reported no change of mold exposure profiles since the initial sampling based on our published questionnaire [33]. Colored boxes indicate the maximum range allowing for a CV of 15% (light grey) and 25% for defined protein antigens (Aspf4) or 35% for the lysate [32], respectively (dark grey). Abbreviations: AfuLy = *A. fumigatus* mycelial lysate, CV = coefficient of variation, D = donor, inter-oper. = inter-operator, intra-indiv. = intra-individual, Med. = median, WB = whole blood.

Assay precision is a crucial performance indicator for cellular biomarkers [38]. Therefore, we evaluated the CVs of T-cellular cytokine concentrations in two independent stimulation tubes injected by the same operator (technical CV) as well as the concordance of tests conducted by two operators using blood from the same venipuncture (inter-operator CV). Both parameters were very low (median 4.3–13.2%) for IFN-γ release induced by *A. fumigatus* antigens, indicating excellent reproducibility (Figure 3). For IL-17, slightly higher variability was seen; however, median technical and inter-operator CVs (7.7–13.6%) were markedly below commonly accepted thresholds for diagnostic bioassays (Figure 3) [39].

As the intra-individual fluctuation of immune responses to constantly encountered fungal aero-antigens presents an additional source of variation in functional T-cell biomarkers [3,40], we further compared the consistency of *A. fumigatus*-induced IFN-γ and IL-17 secretion in repeated samplings from the same donors after >4 weeks. Considering both antigens and cytokines, the intra-individual CVs ranged from 0.1% to 69.8%, with more than half (10/16) of the antigen/cytokine pairs showing intra-individual CVs below 15% (Figure 3).

### 3.4. Susceptibility to Pre-Analytic Delays

**Figure 4:**
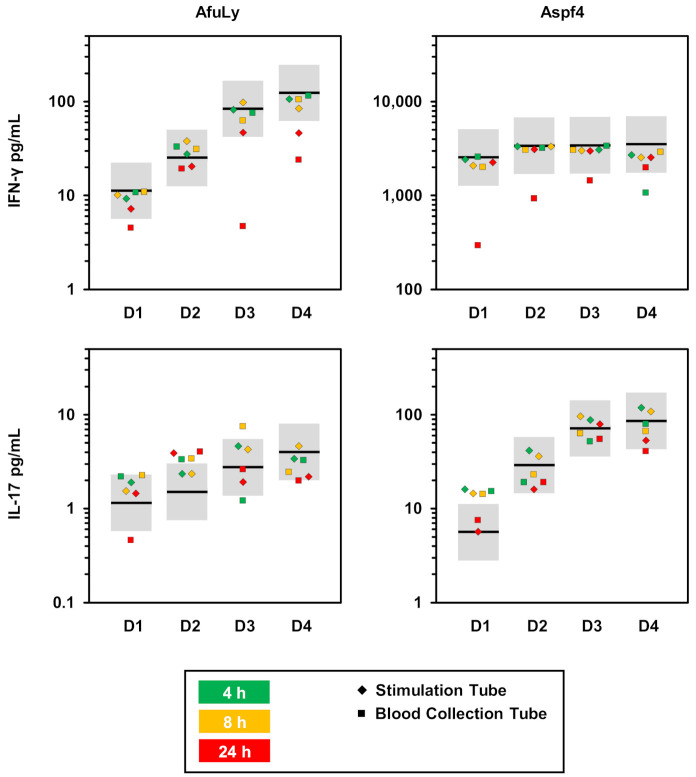
Robustness of WB-ELISA to pre-analytic delays. WB samples from 4 highly mold-exposed healthy donors were stimulated for 24 h with AfuLy or Aspf4 using RPMI-supplemented, α-CD28-, and α-CD49d-containing stimulation tubes, as described in the Materials and Methods Section. IFN-γ and IL-17 concentrations in plasma supernatants were quantified by ELISA. WB was injected and incubated at 37 °C either immediately (black bars) or after pre-analytic storage at room temperature for 4 (green squares), 8 (yellow squares), or 24 h (red squares). In addition, immediately injected stimulation tubes were kept at room temperature for 4 (green diamonds), 8 (yellow diamonds), or 24 h (red diamonds) prior to being transferred to 37 °C for another 24 h incubation period. Individual background-corrected cytokine concentrations for each condition are shown. Grey boxes indicate a ±2-fold change compared with the immediately incubated control sample. Abbreviations: AfuLy = *A. fumigatus* mycelial lysate, WB = whole blood.

Subsequently, we tested the performance of our WB-ELISA protocol after pre-analytic delays, a major limitation of antigen-reactive T-cell assays [13,20,21]. AfuLy- and Aspf4-induced IFN-γ and IL-17 secretion were highly robust after 4 and 8 h storage of injected stimulation tubes at room temperature prior to entering the regular 37 °C incubation cycle, with only 1 out of 16 individual measurements for each time point showing a >2-fold deviation from the immediately processed baseline sample (Figure 4). While leading to slightly greater deviations on average, even 24 h pre-incubation in the stimulation tube resulted in 7 out of 8 valid measurements for each cytokine (Figure 4). In contrast, a considerable number of >2-fold deviations from the baseline measurement were seen, particularly after 24 h, when blood was stored in heparinized blood collection tubes instead of being immediately injected into the stimulation tubes (Figure 4). 

### 3.5. Application of WB-ELISA in Patients with Aspergillus Sensitization

**Figure 5:**
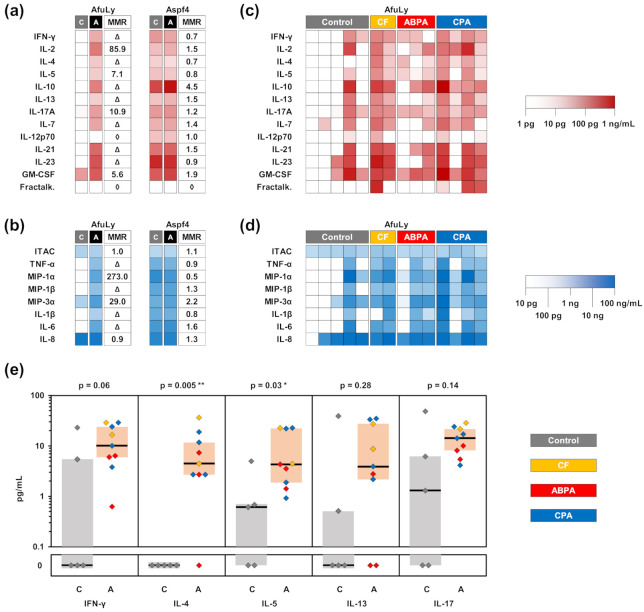
Comparison of *Aspergillus*-induced cytokine release in patients with *Aspergillus*-associated lung pathologies and other chronic lung diseases. WB from 9 patients with *Aspergillus*-associated lung pathologies (2 CF with *A. fumigatus* sensitization, 3 ABPA, and 4 CPA) and a control group of 5 patients with other chronic lung diseases was stimulated with AfuLy or Aspf4 using the RPMI-supplemented, α-CD28-, and α-CD49d-containing stimulation tubes, as described in the Materials and Methods Section. Cytokine concentrations in plasma supernatants were quantified using a 21-plex Luminex assay. (**a**,**b**) Heat maps indicating median background-adjusted cytokine concentrations in the ABPA/CF/CPA patient cohort “A” and control cohort “C” depending on the antigen used for stimulation. The numeric value in the MMR column represents the median-to-median ratio between the two cohorts, with values > 1.0 indicating greater median cytokine concentrations in the ABPA/CF/CPA cohort. Δ denotes infinite median-to-median ratios (median = 0 pg/mL in the control cohort). ◊ denotes undefined median-to-median ratios (median = 0 pg/mL in both cohorts). (**c**,**d**) Heat maps summarizing individual cytokine response to AfuLy in all 14 patients. (**e**) Comparison of individual AfuLy-induced concentrations of selected T-helper cell signature cytokines. Medians and interquartile ranges for patients with ABPA/CF/CPA (“A”, red) and controls (“C”, grey) are indicated by black bars and colored boxes, respectively. Two-sided Mann–Whitney U test. Abbreviations: AfuLy = *A. fumigatus* mycelial lysate, ABPA = allergic bronchopulmonary aspergillosis, CF = cystic fibrosis, CPA = chronic pulmonary aspergillosis, Fractalk. = fractalkine, WB = whole blood.

We then sought to evaluate the performance of WB-ELISA in patients with *Aspergillus*-associated lung pathologies that are etiologically linked to an aberrant cytokine milieu, disproportionate T-cell activation, and pathologically elevated IL-17 secretion [1,41,42,43]. To that end, we tested the capability of the assay to discriminate cytokine concentrations in antigen-stimulated WB samples taken from patients with cystic fibrosis (CF), allergic bronchopulmonary aspergillosis (ABPA), or chronic pulmonary aspergillosis (CPA), and control patients who had non-mold-related chronic interstitial lung diseases (Figure 5). 

We compared WB stimulation with AfuLy and the proteinaceous *A. fumigatus* antigen Aspf4 and assessed plasma supernatants with a 21-plex cytokine assay (Figure 5a,b). The lysate provided a stronger discriminatory power between the two cohorts than Aspf4, with median-to-median ratios of at least 7.1 for all T-helper cell signature cytokine responses tested (Figure 5c). Several T-cellular signature cytokines displayed non-overlapping interquartile ranges of concentrations in AfuLy-stimulated samples from patients with *Aspergillus*-associated diseases and controls (Figure 5e). In addition to an increased IL-17 response to the lysate in CF/ABPA/CPA patients (median, 14.3 versus 1.3 pg/mL in controls), significantly greater induction of the Th2 cytokines IL-4 and IL-5 was found in samples from patients with *Aspergillus*-associated lung diseases (Figure 5e). This observation was corroborated by a trend toward elevated IL-13 levels, another Th2 cytokine linked to allergic airway inflammation [44,45], in samples from CF/ABPA/CPA patients. APC-derived cytokines such as MIP-1α, MIP-1β, MIP-3α, IL-1β, and IL-6 were also strongly upregulated in AfuLy-stimulated blood from patients with *Aspergillus*-associated lung pathologies compared to controls (Figure 5b,d). In contrast, Aspf4 failed to discriminate T-cellular and APC-derived cytokine endpoints between the two cohorts (Figure 5a,b). Background-adjusted raw data for all patients and both stimuli are provided in Supplementary Tables S2 (AfuLy) and S3 (Aspf4).

## 4. Discussion

There is increasing interest in WB-based profiling of adaptive immune responses in infectious diseases and, to that end, a number of investigational and commercial assay systems have been developed [17,46]. WB assays for the detection of antigen-reactive T-cells have several important advantages over their PBMC-based counterparts as they require less blood volume and hands-on-time, facilitate improved standardization, and are often more cost-efficient [47,48,49]. WB also provides a more physiological representation of the in vivo environment, since individual immune cell ratios, granulocytes, and platelets are preserved [47,48]. Of note, granulocytes are important activators of secondary stimulation loops and are known to shape phenotypes of adaptive cellular immunity [50]. Accordingly, we and others reported greater stimulatory capacity and improved reproducibility of WB-based protocols compared with conventional PBMC assays [16,47,49,50]. The present study corroborated these observations by providing direct evidence for T-cell-dependent granulocyte activation and release of APC-derived cytokines in our WB-ELISA stimulation tubes (Figure 2c,d).

Mold-reactive T-cell assays tend to be particularly susceptible to suboptimal stimulation conditions [11,12], likely due to the heterogeneity of reactive T-cell subsets, complex antigenic stimuli, and pivotal contributions of Th2 and Th17 cells [1], which are less robust and demonstrate delayed activation kinetics [51]. To tackle these challenges, we recently highlighted the crucial role of enhanced co-stimulation to improve the efficacy and robustness of flow cytometric mold-reactive T-cell detection, especially after exposure to immunosuppressive agents [16,23]. In line with these reports, α-CD28 addition noticeably yet non-significantly boosted cytokine secretion in our WB-ELISA system, whereas dual co-stimulation with both α-CD49d and α-CD28 was essential to maximize A. *fumigatus*-reactive IFN-γ and IL-17 release. These findings were confirmed by magnetic multiplex cytokine profiling, which revealed improved detection rates for most of the studied T-cellular cytokines as well as various APC cytokines (e.g., IL-1β and MIP-1α), suggesting that (dual) co-stimulation also enhances T-cell/APC feedback loops.

Although CD28 and CD49d can drive T-cell activation and viability, enhance cytokine secretion, and improve assay performance [29,30,52,53,54,55], to our knowledge, none of the commercial IGRAs routinely use co-stimulatory antibodies, likely due to concerns over non-specific background reactivity. However, concordant with our flow cytometry-based experience [16] and other reports [53,56], non-specific background secretion of T-cellular cytokines was minimal in our optimized ELISA system and remained unchanged with dual co-stimulation.

Technical and inter-operator variation of WB-ELSA was low and within commonly accepted thresholds for cell-based bioassays [32,39]. Additionally, the WB-ELISA protocol showed superior robustness to pre-analytic delays compared with previously published data for PBMC-based assays (including ELISA) to detect *A. fumigatus*-specific T-cell activation [12,13]. A recent study by our consortium further revealed an overall promising performance of WB-ELISA in allogenic hematological stem cell transplant recipients [57]. Specifically, high technical reliability and good concordance of IFN-γ response to CMV phosphoprotein 65 with CMV serostatus and reactivation was seen in patients with >800 lymphocytes per µL of peripheral blood. Combined with the present study, these results indicate high assay robustness using antigens with varying complexity and different T-cell activation kinetics.

Importantly, blood samples stored in conventional heparinized blood collection tubes prior to injection were more susceptible to long pre-analytic delays, underscoring a protective role of medium supplementation and dual co-stimulation to maintain T-cell viability and functionality. Consequently, blood should be injected into the stimulation system immediately after the blood draw, a process that is similar to the injection of blood culture flasks [16]. The injected stimulation tubes could then be transported to adequate laboratory facilities at room temperature during an 8-h window, if onsite incubation or temperature-controlled transportation are not available. 

While evaluation of the diagnostic performance in invasive aspergillosis is needed in future, sufficiently powered multi-center studies, we applied our optimized WB-ELISA to patients with a spectrum of chronic *Aspergillus*-associated lung pathologies that frequently lead to altered T-cell response to mold antigens and enhanced release of Th2 and Th17 signature cytokines. Specifically, increased IL-17 response has been associated with chronic lung diseases that predispose to airway colonization by molds including *Aspergillus* [58,59] and can promote fungal persistence and tissue inflammation [1,60]. Accordingly, our WB-ELISA assay revealed increased, AfuLy-induced, IL-17 release in most patients with CF, ABPA, or CPA. Although statistical significance was not reached for IL-17 due to the small sample size, our finding of an over 10-fold median-to-median ratio and non-overlapping interquartile ranges suggests high discriminatory power of the optimized ELISA assay that could surpass the discriminatory potential of intracellular IL-17 staining [58,61]. 

Interestingly, our results also indicated notable elevation of Th2 cytokines IL-4, IL-5, and IL-13 in ABPA/CF/CPA patients versus controls. This observation is consistent with a murine study suggesting that chronic or repeated exposure to *Aspergillus* induces co-evolution of Th1, Th2, and Th17 cells [62]. Furthermore, PBMCs from patients with acute ABPA stimulated with *Aspergillus* antigens have been reported to show enhanced Th2 cytokine response, resulting in an increased Th2/Th1 ratio [63,64]. However, in line with earlier reports [3,33,58,65], considerable induction of T-helper cell signature cytokines by AfuLy was also identified in some of our control patients (Figure 5E) and healthy subjects with frequent mold exposure (Figure 1, Figure 2, Figure 3 and Figure 4). Future studies would be warranted to determine the usefulness of WB-ELISA to track occupational or residential mold exposure and to elucidate the influence of environmental mold exposure on the assay’s diagnostic performance in both invasive mold infections and hypersensitivity syndromes.

Our study had several limitations. Firstly, the only commercially available T-cell-optimized, research-grade *A. fumigatus* lysate (Miltenyi Biotec) was recently discontinued, and the in-house lysate does not provide comparably consistent stimulation efficiency. Differences in stimulation capacity were also observed amongst different batches of Aspf4 antigens. Although this limits comparability between experimental series, it does not represent an inherent limitation of the WB-ELISA technique per se. However, commercial availability of quality-controlled and cost-efficient mold antigens would be essential in order to improve the standardization of functional T-cell assays across different laboratories. Secondly, our enhanced WB-ELISA assay displayed heightened sensitivity to residual endotoxins in antigen preparations. This necessitated enhanced, industry-grade purification of antigenic stimuli in order to reduce the residual endotoxin content below 1 endotoxin unit per mg of antigen and may limit the selection of applicable infection-related antigens for future studies. Thirdly, as is the case for blood culture bottle inoculation, this assay requires a strictly aseptic venipuncture technique and thorough disinfection of the membrane of the stimulation tube, since the test is susceptible to microbial contamination. Lastly, the patient cohort for validation of the assay in the present study was limited in size and did not cover patients with proven or probable invasive aspergillosis due to a lack of appropriate samples. Nonetheless, the technical reliability and high discriminatory power observed in the present study and the solid performance of our WB-ELISA system in allogenic stem cell transplant recipients [57] encourage future multi-center evaluation in immunocompromised patients.

## 5. Conclusions

We have developed a cost-efficient, small-volume WB-ELISA system with enhanced co-stimulation that facilitates robust yet specific detection of T-helper cell signature cytokines and T-cell-dependent APC activation in response to *Aspergillus* antigens. Our results indicate good reproducibility and resilience to pre-analytic delays of up to 8 h. Our proof-of-concept study demonstrates high concordance of *Aspergillus*-reactive cytokine signatures with the etiology of chronic lung pathologies. Collectively, these data encourage the future evaluation of WB-ELISA as a platform to develop supportive immune surveillance strategies for opportunistic infections or mold-reactive hypersensitivity syndromes.

## Figures and Tables

**Table 1:** Preparation of stimulation tubes for WB stimulation.

	α-CD28	α-CD49d	AfuLy	Aspf4	PHA	RPMI
**Concentration in the ready-to-use stimulation tube**	2 µg/mL	2 µg/mL	100 µg/mL	60 µg/mL	10 µg/mL	ad 500 µL
**Final concentration afterinjection of 500 µL WB**	1 µg/mL	1 µg/mL	50 µg/mL	30 µg/mL	5 µg/mL	n/a
**Unstimulated control**	X	X				X
**AfuLy stimulation**	X	X	X			X
**Aspf4 protein stimulation**	X	X		X		X
**Positive control**					X	X

X indicates that the compound was used for the respective condition. Abbreviations: AfuLy = *A. fumigatus* mycelial lysate, CD = cluster of differentiation, n/a = not applicable, PHA = phytohemagglutinin, RPMI = Roswell Park Memorial Institute medium, WB = whole blood.

## Data Availability

The data presented in this study are available on request from the corresponding authors.

## Supplementary Materials

The following are available online at https://www.mdpi.com/article/10.3390/jof7060462/s1. Table S1: Antibodies used for flow cytometric studies. Table S2: Raw data for AfuLy-induced cytokine release (in pg/mL) in samples from patients with *Aspergillus*-associated lung pathologies and other chronic lung diseases. Table S3: Raw data for Aspf4-induced cytokine release (in pg/mL) in samples from patients with *Aspergillus*-associated lung pathologies and other chronic lung diseases.
