# Supplementary material for: Development of a Simple and Robust Whole Blood Assay with Dual Co-Stimulation to Quantify the Release of T-Cellular Signature Cytokines in Response to Aspergillus fumigatus Antigens
Source: J Fungi (Basel). 2021 Jun 8;7(6):462. doi: 10.3390/jof7060462 (PMC8230040; doi:10.3390/jof7060462)
Supplement: Supplementary file 1 [file jof-07-00462-s001.zip › jof-1210611-supplementary.pdf]

Development of a Simple and Robust Whole Blood Assay with Dual Co-Stimulation to Quantify the Release of T-Cellular Signature Cytokines in Response to *Aspergillus Fumigatus* Antigens

Supplement

Table S1: Antibodies used for flow cytometric studies.

Fluorochrome		Panels			
		Cell populations	T-cell activation	Granulocyte activation	NK-cell activation
**VioBlue**	**Marker**	CD3	CD3		
	**Volume**	2 µl	2 µl		
	**Company**	Miltenyi Biotec	Miltenyi Biotec		
**FITC**	**Marker**	CD66b	CD4	CD66b	CD3
	**Volume**	2 µl	2 µl	2 µl	10 µl
	**Company**	Miltenyi Biotec	Miltenyi Biotec	Miltenyi Biotec	BD
**PE**	**Marker**		IFN-ɣ	CD62L	IFN-ɣ
	**Volume**		2 µl	2 µl	2 µl
	**Company**		Miltenyi Biotec	Miltenyi Biotec	Miltenyi Biotec
**PE-Vio615**	**Marker**		CD69		CD69
	**Volume**		2 µl		2 µl
	**Company**		Miltenyi Biotec		Miltenyi Biotec
**PerCP**	**Marker**	CD14	CD8		CD16
	**Volume**	2 µl	2 µl		5 µl
	**Company**	Miltenyi Biotec	Miltenyi Biotec		Biolegend
**PE-Vio770**	**Marker**		CD107a		CD107a
	**Volume**		10 µl		10 µl
	**Company**		Miltenyi Biotec		Miltenyi Biotec
**APC**	**Marker**	CD56	CD154	CD11b	CD56
	**Volume**	5 µl	2 µl	2 µl	5 µl
	**Company**	BD	Miltenyi Biotec	Miltenyi Biotec	BD
**APC-Vio750**	**Marker**	L/D fixable near-IR	L/D fixable near-IR	L/D fixable near-IR	L/D fixable near-IR
	**Volume**	0.1 µl	0.1 µl	0.1 µl	0.1 µl
	**Company**	Invitrogen	Invitrogen	Invitrogen	Invitrogen

Added directly to WB stimulation tubes as described in Materials & Methods (section 2.7)

Life/Dead staining (L/D) performed before extracellular staining in 1 ml Hank's balanced salt solution for 30 min

Extracellular staining

Intracellular staining

Abbreviations: APC = allophycocyanin, FITC = fluorescein isothiocyanate, IR = infra red, PE = phycoerythrin, PerCP = Peridinin-Chlorophyll-Protein.

Table S2: Raw data for AfuLy-induced cytokine release (in pg/ml) in samples from patients with *Aspergillus*-associated lung pathologies and other chronic lung diseases.

Patient →	Control	Control	Control	Control	Control	CF	CF	ABPA	ABPA	ABPA	CPA	CPA	CPA	CPA	Median	Median	MMR
**Cytokine**	#1	#2	#3	#4	#5	#1	#2	#1	#2	#3	#1	#2	#3	#4	**Controls**	**Asp-ass Dis.**	
**IFN-γ**	0.0	0.0	0.0	23.2	5.5	28.9	16.7	6.4	6.0	0.6	29.0	3.8	23.9	10.0	0.0	10.0	INF
**IL-2**	0.0	0.4	0.0	136.2	1.0	46.4	4.8	0.8	4.2	36.1	46.1	41.7	312.6	4.0	0.4	36.1	85.9
**IL-4**	0.0	0.0	0.0	0.0	0.0	36.5	4.5	7.3	0.0	2.7	19.0	2.7	11.7	2.7	0.0	4.5	INF
**IL-5**	0.0	0.0	0.6	5.0	0.7	22.6	4.5	3.6	1.4	4.3	22.2	0.9	22.6	1.9	0.6	4.3	7.1
**IL-10**	0.0	0.0	0.0	292.6	0.0	185.5	70.4	0.0	20.3	72.8	1886.9	10.8	18.7	32.4	0.0	32.4	INF
**IL-13**	0.5	0.0	0.0	39.3	0.0	27.4	8.7	0.0	0.0	2.8	33.5	2.2	34.8	3.9	0.0	3.9	INF
**IL-17A**	0.0	0.0	1.3	48.3	6.2	28.7	21.6	8.2	10.1	5.5	24.1	4.1	17.1	14.2	1.3	14.2	10.9
**TNF-α**	2.1	0.0	0.0	2339.4	0.0	561.2	3324.0	0.0	1422.2	158.8	15241.4	5.4	721.4	640.6	0.0	640.6	INF
**MIP-1α**	8.6	0.0	11.6	97207.3	459.9	8137.0	28744.2	45.3	3169.3	1483.2	97207.3	171.5	97191.7	1290.6	11.6	3169.3	273.0
**MIP-1β**	0.0	0.0	0.0	1593.4	0.0	622.8	1026.9	0.0	412.5	415.7	23121.0	175.7	2867.8	587.1	0.0	587.1	INF
**MIP-3α**	0.0	0.0	190.2	14286.5	1939.0	3459.0	12636.9	21.0	10155.0	9671.1	161434.3	256.4	3322.4	5507.9	190.2	5507.9	29.0
**IL-1β**	0.0	0.0	0.0	649.7	4.6	0.0	7716.3	0.0	1527.9	85.9	53147.3	0.0	209.8	1070.2	0.0	209.8	INF
**IL-6**	0.0	0.0	0.0	25229.5	8.2	4647.9	2684.6	2.2	3077.8	3247.5	26841.8	7.0	11703.5	2294.6	0.0	3077.8	INF
**IL-7**	0.0	5.3	0.0	30.1	0.0	56.4	15.0	0.0	0.0	11.8	61.1	9.7	26.0	0.0	0.0	11.8	INF
**IL-8**	0.0	11402.0	52040.8	75918.0	77693.0	36622.0	96147.0	13346.0	57674.0	46756.0	89414.0	7642.5	98065.9	42691.6	52040.8	46756.0	0.9
**IL-12p70**	0.0	0.0	0.0	2.3	0.0	9.1	0.0	0.0	0.0	0.0	4.3	0.0	0.0	1.5	0.0	0.0	ND
**IL-21**	0.0	0.0	0.0	126.7	0.0	129.8	89.5	0.0	17.0	58.3	179.1	0.0	128.8	38.8	0.0	58.3	INF
**IL-23**	0.0	0.0	36.7	146.6	0.0	527.4	180.9	0.0	0.0	37.1	253.4	0.0	169.6	104.0	0.0	104.0	INF
**GM-CSF**	0.0	0.0	15.3	463.4	20.2	167.0	126.6	13.6	85.7	53.5	1204.5	27.1	251.0	42.8	15.3	85.7	5.6
**Fractalkine**	0.0	0.0	0.0	0.0	0.0	356.5	0.0	0.0	0.0	0.0	2.8	0.0	146.6	96.5	0.0	0.0	ND
**ITAC**	116.0	162.7	199.0	347.0	205.0	396.3	531.6	136.0	187.0	208.7	174.6	530.7	324.2	123.0	199.0	208.7	1.0

Abbreviations: ABPA = allergic bronchopulmonary aspergillosis, AfuLy = *A. fumigatus* mycelial lysate, Asp-ass. Dis. = *Aspergillus*-associated diseases, CF = cystic fibrosis, CPA = chronic pulmonary aspergillosis, INF = infinite (0 pg/ml in the control cohort), MMR = median-to-median ratio, ND = not defined (0 pg/ml in both cohorts).

Table S3: Raw data for Aspf4-induced cytokine release (in pg/ml) in samples from patients with *Aspergillus*-associated lung pathologies and other chronic lung diseases.

Patient →	Control	Control	Control	Control	Control	CF	CF	ABPA	ABPA	ABPA	CPA	CPA	CPA	CPA	Median	Median	MMR
**Cytokine**	#1	#2	#3	#4	#5	#1	#2	#1	#2	#3	#1	#2	#3	#4	Controls	Asp-ass Dis.	
**IFN-γ**	17.9	16.8	11.7	15.0	19.1	70.2	8.2	22.7	11.3	0.0	74.9	2.0	8.2	27.1	16.8	11.3	0.7
**IL-2**	7.9	5.8	1.5	0.6	8.5	17.1	8.5	9.6	12.5	3.5	11.0	0.2	4.2	6.0	5.8	8.5	1.5
**IL-4**	3.6	7.3	0.0	0.0	3.6	21.8	2.7	22.0	10.9	0.0	0.0	0.0	0.0	2.7	3.6	2.7	0.7
**IL-5**	5.5	4.8	1.2	6.3	8.6	21.8	3.1	10.3	8.8	0.1	11.3	1.2	2.4	4.6	5.5	4.6	0.8
**IL-10**	83.8	140.9	165.1	183.7	131.1	1244.0	871.9	127.1	631.8	125.5	16510.9	0.0	16.0	1205.6	140.9	631.8	4.5
**IL-13**	5.8	5.1	1.7	2.2	8.8	18.3	7.7	7.8	10.9	0.0	13.8	0.0	7.0	9.8	5.1	7.8	1.5
**IL-17A**	20.1	17.4	1.3	1.3	16.3	31.2	24.6	20.2	21.5	0.0	32.6	0.0	7.5	18.8	16.3	20.2	1.2
**TNF-α**	3821.2	1588.2	1549.4	458.8	5855.0	4319.2	1358.0	733.5	2317.2	172.1	6855.4	5.8	1349.4	3030.4	1588.2	1358.0	0.9
**MIP-1α**	16981.2	917.1	4956.8	29011.3	21490.8	17247.0	23817.2	5772.5	3619.3	2302.2	97207.3	192.1	97191.7	8383.6	16981.2	8383.6	0.5
**MIP-1β**	3165.2	233.1	11608.6	1901.4	4591.6	1482.8	4757.9	4294.9	1008.3	3069.1	23121.0	377.3	7902.8	4026.1	3165.2	4026.1	1.3
**MIP-3α**	2732.5	0.0	2008.1	1435.5	6138.0	1963.0	11105.9	2249.3	5514.0	4790.1	51071.3	448.4	1423.4	4409.9	2008.1	4409.9	2.2
**IL-1β**	2346.7	544.3	873.1	2833.3	4274.9	3334.8	805.8	1786.8	1531.9	189.3	30955.3	6.0	2881.3	3316.2	2346.7	1786.8	0.8
**IL-6**	7674.8	1403.3	4808.5	23183.5	8457.1	11960.9	4989.6	5462.0	14414.8	2043.5	26208.8	88.3	15881.5	12362.6	7674.8	11960.9	1.6
**IL-7**	12.7	3.9	1.4	NA	57.1	28.0	0.0	0.0	62.1	11.4	57.3	0.0	36.3	0.0	8.3	11.4	1.4
**IL-8**	22731.0	624.0	7935.8	24880.0	57937.0	28772.0	48113.0	17375.0	44006.0	8148.0	101520.0	289.5	74112.9	19525.6	22731.0	28772.0	1.3
**IL-12p70**	14.0	2.2	0.0	5.5	16.2	25.0	0.0	5.5	7.2	0.0	6.7	0.0	2.3	8.5	5.5	5.5	1.0
**IL-21**	34.2	44.0	35.9	81.0	65.5	97.1	49.5	0.0	136.5	19.6	172.8	0.0	133.6	66.2	44.0	66.2	1.5
**IL-23**	141.6	351.2	109.5	468.4	883.0	789.4	127.0	353.7	300.1	37.1	360.4	0.0	527.7	157.0	351.2	300.1	0.9
**GM-CSF**	79.9	85.4	53.5	168.5	137.8	311.1	87.6	159.4	184.0	33.4	759.2	0.0	204.6	97.6	85.4	159.4	1.9
**Fractalkine**	0.0	167.5	0.0	0.0	174.2	253.6	0.0	0.0	155.4	0.0	0.0	0.0	0.0	112.3	0.0	0.0	ND
**ITAC**	254.0	24.6	6.0	147.0	327.0	306.8	186.6	155.7	188.0	9.5	101.6	155.7	20.6	10.9	147.0	155.7	1.1

Abbreviations: ABPA = allergic bronchopulmonary aspergillosis, AfuLy = *A. fumigatus* mycelial lysate, Asp-ass. Dis. = *Aspergillus*-associated diseases, CF = cystic fibrosis, CPA = chronic pulmonary aspergillosis, INF = infinite (0 pg/ml in the control cohort), MMR = median-to-median ratio, NA = not available (invalid measurement), ND = not defined (0 pg/ml in both cohorts).
